# High expression of p300 is linked to aggressive features and poor prognosis of Nasopharyngeal Carcinoma

**DOI:** 10.1186/1479-5876-10-110

**Published:** 2012-05-30

**Authors:** Zhi-Wei Liao, Tong-Chong Zhou, Xiao-Jun Tan, Xian-Lu Song, Yuan Liu, Xing-Yuan Shi, Wen-Jin Huang, Li-Li Du, Bo-Jun Tu, Xiao-dan Lin

**Affiliations:** 1 Department of Radiation Oncology, Affiliated Tumor Hospital of Guangzhou Medical College, No.78 Hengzhigang Road Yuexiu District, Guangzhou 510095, China; 2 Department of Pathology, Affiliated Tumor Hospital of Guangzhou Medical College, No.78 Hengzhigang Road, Yuexiu District, Guangzhou 510095, China

**Keywords:** Nasopharyngeal carcinoma, p300, Immunohistochemistry, Prognosis

## Abstract

**Background:**

Increased expression of transcriptional coactivator p300 has been observed in a variety of human cancers. However, the expression status of p300 protein/mRNA in nasopharyngeal carcinoma (NPC) tissues and its clinicopathologic/prognostic implication are poorly understood.

**Methods:**

In our study, mRNA and protein expression levels of p300 was explored by reverse transcription-polymerase chain reaction (RT-PCR), Western blotting (WB) and immunohistochemistry (IHC) in nasopharyngeal mucosal and NPC tissues. The data were analyzed by receiver operating characteristic (ROC) curve analysis, spearman’s rank correlation, Kaplan-Meier plots and Cox proportional hazards regression model.

**Results:**

Up-regulated expression of *p300* mRNA/p300 protein was detected in NPC tissues by RT-PCR and WB, when compared to nasopharyngeal mucosal tissues. Based on ROC curve analysis, the cutoff score for p300 high expression was defined when more than 35% of the tumor cells were positively stained. High expression of p300 was observed in 127/209 (60.7%) of NPCs. In NPCs, high expression of p300 was positively associated with later T classification, later N classification, distant metastasis and later clinical stage (*P* < 0.05). In univariate survival analysis, overexpression of p300 was found to be an indicator of progression-free (*P* = 0.002) and overall survival (*P* = 0.001) in NPCs. More importantly, p300 expression was evaluated as an independent prognostic factor for NPC in multivariate analysis (*P* = 0.036).

**Conclusions:**

Our findings support that high expression of p300 protein might be important in conferring a more aggressive behavior, and is an independent molecular marker for shortened survival time of patients with NPC.

## Background

Nasopharyngeal carcinoma (NPC) is a common malignancy in southern China. The etiologic factors associated with NPC are believed to be genetic susceptibility, EBV infection, and other environmental factors [1-3]. Like other types of human malignant tumors, a multi-step process consisting of initiation, local progression and metastasis appears to be dictated by a wide variety of abnormal genetic changes and this process is involved in the development/progress of NPC [1]. However, the precise genetic changes that are responsible for NPC progression are largely unknown. Thus, the identification of novel genetic biomarkers is of paramount importance because this would allow early detection of this cancer, provide new therapeutic targets for treatment and ultimately improve overall survival for patients with NPC.

p300, which is known as KAT3B/EP300, is a member of the histone acetyltransferase family of transcriptional coactivator. p300 is found to play a variety of roles in the transcription process and catalyzes histone acetylation through its histone acetyltransferase activity [4,5]. Transcriptional coactivator p300 is involved in the regulation of various cellular processes such as proliferation, differentiation, apoptosis, cell-cycle regulation and DNA damage response [6]. Previous studies also suggested that p300 played a tumor suppressor role in certain human malignancies, including breast, colorectal and gastric carcinoma [7,8]. However, recent studies suggest that p300 is a positive regulator of cancer progression and related to tumorigenesis of various cancers [9-11]. Marije et al. indicated that p300 was a cofactor correlated with p53 accumulation and HIF-1a levels in invasive breast cancer [12]. It has also been reported that p300 expression correlates with nuclear alterations of tumor cells and contributes to the growth of prostate carcinoma and is a predictor of aggressive features of this cancer [13,14].

Up to date, the expression dynamics of p300 in NPCs and its clinicopathologic/prognostic significance has not been elucidated. In the present study, RT-PCR and Western blotting, tissue microarray and immunohistochemistry (IHC) were employed to examine the distribution and frequency of p300 mRNA/protein expression in our NPC and nasopharyngeal mucosal tissues. The correlations between p300 expression and patient clinicopathologic and prognostic significance were assessed to determine whether the expression of p300 could be a prognostic biomarker for patients with NPC.

## Methods

### Patients and tissue specimens

In this study, the paraffin-embedded pathologic specimens from 209 patients with NPC were obtained from the archives of Department of Pathology, affiliated Tumor Hospital of Guangzhou Medical College, Guangzhou, China, between February 2004 and July 2007. These NPC cases included 150 (71.8%) men and 59 (28.2%) women, with mean age of 45 years. Average follow-up time was 72.8 months (range, 3.0 to 233 months). Clinicopathologic characteristics for these patients including age, sex, Histological classification, T classification, N classification, distant metastasis, Clinical stage were described in Table1. For the use of these clinical materials for research purposes, prior patient’s consent and approval from the Institute Research Ethics Committee were obtained. The disease stages of all the patients were classified or reclassified according to the 1992 nasopharyngeal carcinoma staging system of China**.**

**Table 1:** Correlation between the expression of P300 and clinicopathologic features in nasopharyngeal carcinomas

		**P300 protein**		
	**All cases**	**Low expression**	**High expression**	*P*value ^*^
Sex				0.560
Female	59	25 (42.4%)	34 (57.6%)	
Male	150	57 (38.0%)	93 (62.0%)	
Age at diagnosis (years)				0.583
≤ 45	89	33 (37.1%)	56 (62.9%)	
> 45	120	49 (40.8%)	71 (59.2%)	
Histological classification (WHO)				0.363
Type II	54	24 (44.4%)	30 (55.6%)	
Type III	155	58 (37.4%)	97 (62.6%)	
T classification				0.000
1	26	19 (73.1%)	7 (26.9%)	
2	68	37 (54.4%)	31 (45.6%)	
3	69	17 (24.6%)	52 (75.4%)	
4	46	9 (19.6%)	37 (80.4%)	
N classification				0.024
0	40	23 (57.5%)	17 (42.5%)	
1	96	34 (35.4%)	62 (64.6%)	
2	51	15 (29.4%)	36 (70.6%)	
3	22	6 (27.3%)	16 (72.7%)	
Distant metastasis				0.021
0	158	69 (43.7%)	89 (56.3%)	
1	51	13 (25.5%)	38 (74.5%)	
Clinical stage				0.000
I	10	8 (80.0%)	2 (20.0%)	
II	55	37 (67.3%)	18 (32.7%)	
III	83	23 (27.7%)	60 (72.3%)	
IV	61	14 (23.0%)	47 (77.0%)	

^*^Chi-square test; WHO, World Health Organization.

### Reverse transcription-polymerase chain reaction (RT-PCR)

Total RNA was isolated from 4 pairs of NPC and nonneoplastic nasopharyngeal mucosal tissues using TRIZOL reagent (Invitrogen, Carlsbad, CA). RNA was reverse-transcribed using SuperScript First Strand cDNA System (Invitrogen, Carlsbad, CA) according to the manufacture’s instructions. The following primers were used for amplification of p300: sense primer 5'-AAACCCACCAGATGAGGA C-3', antisense primer, 5'-TATGCACTAGATGGCTCCGCAG-3'. The primers used for beta actin (internal control) were 5'-CGAAACTACCTTCAACTCCATCA-3' and 5'-CGGACTCGTCATACTCCTGCT-3'. The PCR products were analyzed by agarose gel electrophoresis and confirmed by appropriate size and/or sequencing.

### Western blotting analysis

Equal amounts of tissue lysates were separated by SDS-PAGE and electrophoretically transferred to a polyvinylidene difluoride membrane. Membranes were blocked in Tris-buffered saline with 5% milk and 0.05% Tween, and probed with p300 antibody (Abcam, Cambridge, MA) at 4°C overnight. After washing, the membranes were incubated with horseradish peroxidase-conjugated goat anti-mouse secondary antibodies (Jackson ImmunoResearch Laboratories) and visualized using the enhanced chemiluminescence kit (Forevergen Biosciences, China). The procedures followed were conducted in accordance with the manufacturer’s instructions.

### Tissue microarray (TMA) construction

Tissue microarray was constructed as the method described previously [15]. Briefly, tissues samples from 250 NPC and 40 nasopharyngitis cases were collected, fixed in formalin and embedded in paraffin. H&E-stained slides were reviewed by a senior pathologist to determine and mark out representative tumor areas. Duplicates of 0.6 mm diameter cylinders were punched from representative tumor areas of individual donor tissue block and re-embedded into a recipient paraffin block at defined position, using a tissue arraying instrument (Beecher Instruments, Silver Spring, MD). After the exclusion of cores with inadequate tissue after sectioning and tissue transfer, the final IHC analyses included 209 NPC and 30 nasopharyngitis cases.

### Immunohistochemistry (IHC)

The TMA slides were dried overnight at 60°C, deparaffinized in xylene, rehydrated through graded alcohol, immersed in 3% hydrogen peroxide for 20 minutes to block endogenous peroxidase activity, and antigen-retrieved by pressure cooking for 3 minutes in ethylenediamine tetraacetic acid (EDTA) buffer (pH = 8.0). Then the slides were preincubated with 10% normal goat serum at room temperature for 30 minutes to reduce nonspecific reaction. Subsequently, the slides were incubated with mouse monoclonal anti-p300 (Abcam, Cambridge, MA) at a concentration of 3 ng/ml for 2 hours at room temperature. The slides were sequentially incubated with a secondary antibody (Envision; Dako, Glostrup, Denmark) for 1 hour at room temperature, and stained with DAB (3,3-diaminobenzidine). Finally, the sections were counterstained with Mayer’s hematoxylin, dehydrated, and mounted. A negative control was obtained by replacing the primary antibody with a normal mouse IgG.

### IHC evaluation

Nuclear staining for p300 protein was reported in semi-quantitative method by assessing the number of positive cancer and mucosal cells over the total number of cancer and mucosal cells, respectively. Scores were assigned by using 5% increments (0%, 5%, 10%-100%). Expression for p300 was scored by 3 independent pathologists blinded to clinicopathological data. Their conclusions were in complete agreement in 78.5% of the cases, which identified this scoring method as highly reproducible.

### Selection of cutpoint score

ROC curve analysis was employed to determine cutoff score for tumor “high expression” by using the 0, 1-criterion [16]. For the p300 score, the sensitivity and specificity of each outcome under study was plotted, thus generating various ROC curves. The score was selected as the cutoff value, which was closest to the point with both maximum sensitivity and specificity. Tumors designated as “low expression” for p300 were those with scores below or equal to the cutoff value, while “high expression” tumors were those with scores above the value. For ROC curve analysis, the clinicopathologic features were dichotomized: T classification (T1-T2 versus T3-T4), N classification (N0-N1 versus N2-N3), distant metastasis (M0 versus M1), clinical stage (I-II versus III-IV), cancer progression (Yes versus No) and survival status (death due to NPC versus censored).

### Statistical analysis

Statistical analysis was performed by using the SPSS statistical software package (standard version 13.0; SPSS, Chicago, IL). ROC curve analysis was utilized to define the cutoff score for high expression of p300. The correlation between p300 expression and clinicopathological characteristics of NPC patients was evaluated by *χ*^2^-test. Univariate and multivariate survival analyses were performed using the log-rank test and Cox proportional hazards regression model, respectively. Survival curves were obtained with the Kaplan-Meier method. Differences were considered significant if the *P*-value from a two-tailed test was <0.05.

## Results

### The expression of p300 in NPCs and nonneoplastic mucosal tissues by RT-PCR and western blotting

**Figure 1:**
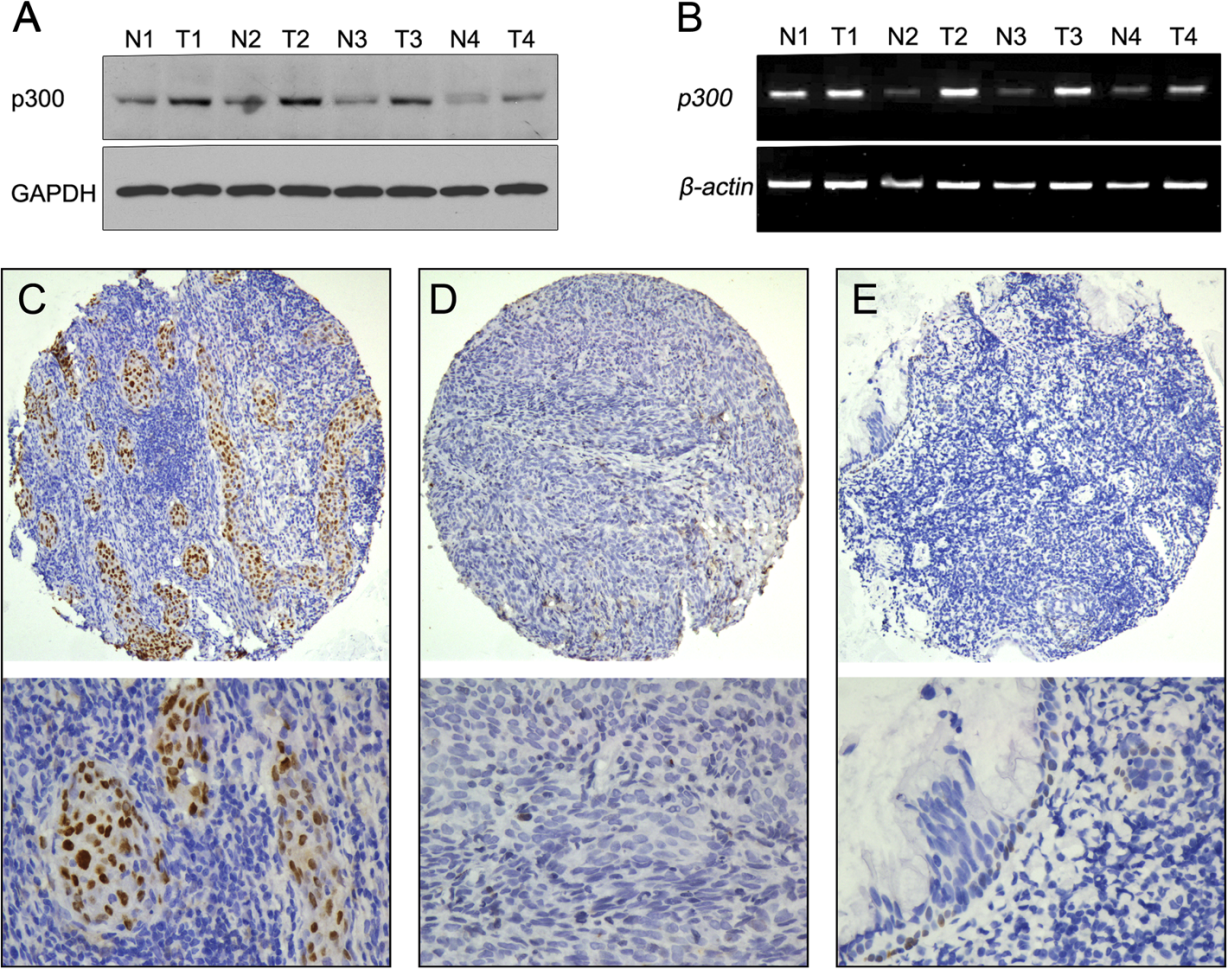
**The mRNA and protein expression of p300 in NPCs and nonneoplastic mucosal tissues.A**. Up-regulated expression of *p300* mRNA was examined by RT-PCR in 4/4 NPC cases, when compared with n nonneoplastic mucosal tissues. **B**. Up-regulated expression of p300 protein was detected by Western blotting in 4/4 HCC cases, when compared with nonneoplastic mucosal tissues. **C**. High expression of p300 was observed in a NPC, in which more than 90% tumor cells revealed positive immunostaining of p300 in nuclei (*upper panel*, ×100). **D**. A NPC case demonstrated low expression of p300, in which less than 5% of tumor cells showed immunoreactivity of p300 protein in nuclei (*upper panel*, ×100). **E**. The nonneoplastic mucosal tissue showed nearly negative expression of p300 protein (*upper panel*, ×100). The *lower panels* indicated the higher magnification (×400) from the area of the box in **C**., **D**., and **E**., respectively.

In the present study, the levels of expression of *p300* mRNA and p300 protein were examined by RT-PCR and Western blotting, respectively, in 4 pairs of fresh NPC and nonneoplastic mucosal tissues. Our results revealed that all NPCs were examined as having up-regulated p300 protein expression (Figure1A), when compared with nonneoplastic mucosal tissues. Up-regulated expression of *p300* mRNA also was observed in all NPCs (Figure1B).

### The expression patterns of p300 in NPCs and nonneoplastic mucosal tissues by IHC

For p300 IHC staining in NPCs and nonneoplastic mucosal tissues, immunoreactivity was primarily seen in the nuclei within tumor and mucosal cells (Figure1C). A negative control demonstrating the specificity of the signal was shown in a breast cancer with negative expression of p300 (Additional file 1: Figure S1). p300 expression could be assessed informatively in 209 NPCs by the TMA constructed previously. The non-informative TMA samples included samples with too few tumor cells (<300 cells per case) and lost samples. Staining intensity of p300 in NPC ranged from 0% to 100% (Figure1C-1E). According to ROC curve analysis, expression percentage for p300 above the cutoff value 35% was defined as high expression, while below or equal to the cutoff value was considered as low expression. In this study, high expression of p300 could be detected in 127/209 (60.7%) of NPCs. and 8/30 (26.7%) of nonneoplastic mucosal tissues, respectively (*P* < 0.05, Fisher’s exact test).

### Selection of cutoff scores for p300 high expression

**Figure 2:**
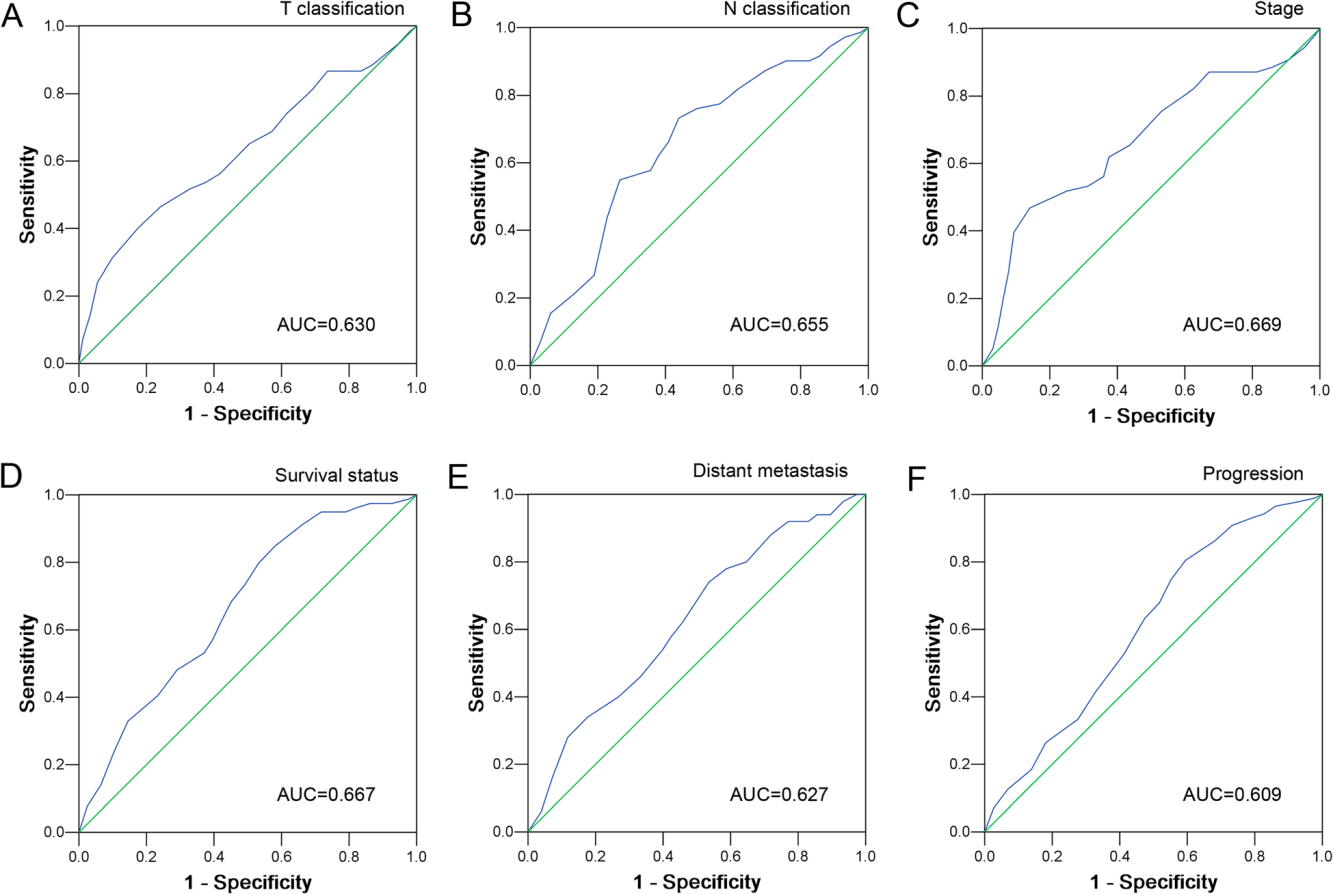
**ROC curve analysis was used to determine the cut-off score for positive expression of p300 protein.** The sensitivity and specificity for each outcome were plotted: (**A**.) T classification (*P* = 0.001); (**B**.) N classification (*P* < 0.001); (**C**.) Clinical stage (*P* < 0.001); (**D**.) Survival status (*P* < 0.001); (**E**.) Distant metastasis (*P* = 0.007); (**F**.) Cancer progression (*P* = 0.008).

The ROC curves for each clinicopathological parameter (Figure2) clearly show the point on the curve closest to (0.0, 1.0) which maximizes both sensitivity and specificity for the outcome as described in previous study [17]. Tumors with scores above the obtained cutoff value were considered as high p300 expression leading to the greatest number of tumors classified based on clinical outcome presence or absence. Cutoff score for p300 high expression was determined to be more than 50% carcinoma cells staining.

### Association of p300 expression with NPC patients’ clinicopathological parameters

The high or low expression rates of p300 in NPCs with respect to several standard clinicopathologic features are presented in Table1. The high p300 expression rate was higher in patients with later T classification (P < 0.0001), later N classification (P = 0.024), distant metastasis (P = 0.021), and later clinical stage (*P* < 0.0001, Table1). There was no significant correlation between p300 expression and other clinicopathologic parameters, such as patient age (≤45 years *vs* >45 years), sex, histological classification (WHO) (*P* > 0.05, Table1).

### Relationship between clinicopathological features, p300 expression, and NPC patients’ survival: Univariate survival analysis

**Figure 3:**
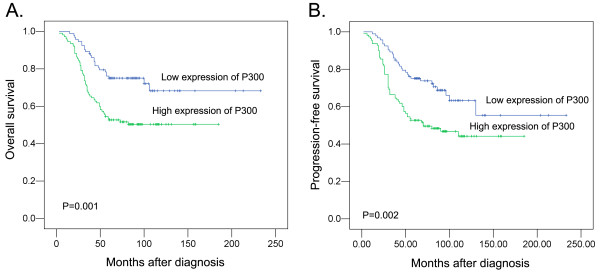
**Kaplan–Meier survival analysis according to p300 expression in 209 patients with nasopharyngeal carcinomas (log-rank test).A**. Relationship of p300 expression to overall survival: low expression, n = 82; high expression, n = 127. **B**. Relationship of p300 expression to progression-free survival: low expression, n = 82; high expression, n = 127.

In order to confirm the representativeness of the NPCs in our study, we analyzed established prognostic factors of patients’ survival. Kaplan-Meier analysis demonstrated a significant impact of well-known clinicopathologic prognostic parameters, such as T classification (*P* = 0.012), N classification (*P* < 0.0001), distant metastasis (*P* < 0.0001), and clinical stage (*P* < 0.0001) on patients’ survival (Table2). Assessment of survival in total NPCs revealed that high expression of p300 was correlated with overall survival (P = 0.001) of NPC patients (Table2, Figure3A). In addition, high expression of p300 in NPCs was evaluated to correlate closely with poor progression-free survival (P = 0.002, Table2, Figure3B).

**Table 2:** Univariate and multivariate analysis of different prognostic variables in 209 patients with nasopharyngeal carcinoma

**Variable**	**Univariate analysis**^**1**^			**Multivariate analysis**^**2**^	
	**All cases**	**Mean survival (months)**	***P*value **	**Hazards ratio (95% CI)**	***P*value **
Sex			0.545		
Female	59	128.9			
Male	150	158.5			
Age at surgery (years)			0.237		
≤ 45	89	119.4			
> 45	120	162.9			
Histological classification (WHO)			0.059		
Type II	54	180.5			
Type III	155	120.3			
T classification			0.012	0.829 (0.444-1.549)	0.557
T1-T2	94	158.5			
T3-T4	115	142.5			
N classification			0.000	1.368 (0.815-2.297)	0.235
N0-N1	136	172.2			
N2-N3	73	79.1			
Distant metastasis			0.000	2.000 (1.217-3.289)	0.006
0	158	171.2			
1	51	69.3			
Clinical stage			0.000	1.836 (0.739-4.563)	0.191
I-II	65	175.7			
III -IV	144	140.0			
P300 expression			0.000	1.830 (1.040-3.220)	0.036
Low	82	172.7			
High	127	135.3			

^1^Log-rank test; ^2^Cox regression model; CI, confidence interval; WHO, World Health Organization.

### Independent prognostic factors of NPC: Multivariate Cox regression analysis

Since features observed to have a prognostic influence by univariate analysis may covariate, p300 expression and those clinicopathologic variables that were significant in univariate analysis (i.e., T classification, N classification, distant metastasis and clinical stage) were further examined in multivariate analysis. Our results showed that high expression of p300 was an independent prognostic factor for poor patient overall survival (hazard ratio, 1.830; 95%CI, 1.040-3.220, *P* = 0.036; Table2). Of the other parameters, Distant metastasis (*P* = 0.006) was evaluated as well independent prognostic factors for patients’ overall survival.

## Discussion

The outcomes of NPC patient with same stage following radiotherapy are different substantially and such large discrepancy has not been investigated. Thus, there is a need for new objective strategies that can effectively distinguish between patients with favorable and unfavorable prognosis for NPC. Molecular biomarkers in conjunction with standard TNM strategy, certainly has the potential to more effectively ascertain patient prognosis and develop appropriate therapeutic regimens. Although NPC has been widely studied, the search and identification of promising molecular and/or genetic alterations in NPC cells that have clinical/prognostic significance remains substantially limited.

p300 was identified initially in protein interaction assays through its correlation with the transcription factor CREB [18]. Transcriptional coactivator p300 has the potential to participate in a variety of cellular functions, including cell proliferation and differentiation, senescence and apoptosis [6]. Recently, several study documented an involvement of p300 in oncogenic processes, such as lung, colon, prostate, breast cancer and leukemia [13,19-22]. In addition, mutation in *p300* gene, accompanied by loss of the other allele, has been observed in certain types of tumors, including colorectal, gastric and breast cancer [7,8]. Up to the present, there is still no study that explored the status of p300 and its potential impact in NPC tumorigenesis.

In the present study, we examined the expression levels of *p300* mRNA and p300 protein in NPC tissues and non-nasopharyngeal carcinoma tissues, firstly by RT-PCR and Western blotting. Our results established that up-regulated expression of *p300* mRNA and p300 protein was shown in the NPCs, when compared to non-nasopharyngeal carcinoma tissues. Subsequently, the expression dynamics of p300 protein was investigated by IHC, using a TMA containing NPC tissues and non-nasopharyngeal carcinoma tissues. Our IHC results demonstrated that high expression of p300 was more frequently observed in NPC tissues than in the non-nasopharyngeal carcinoma tissues. The expression of p300 in non-nasopharyngeal carcinoma tissue was either absent or at low levels. In contrast, in large number of our NPC tissues, high expression of p300 was frequently observed. These findings suggest the possibility that up-regulated expression of p300 may provide a selective advantage in NPC tumorigenic processes.

To assess the significance of p300 protein in NPCs and avoid predetermined arbitrary cutpoint, ROC curve analysis was utilized to determine cut-off score for p300 high expression as described previously [16]. Further correlation analysis showed that high expression of p300 in NPCs was correlated with T classification, N classification, distant metastasis, and clinical stage. More importantly, high expression of p300 was a strong and independent predictor of shortened overall survival as evidenced by univariate and multivariate analysis. Our findings in this study suggest that expression of p300 in NPC may facilitate an increased malignant feature and/or worse prognosis of this tumor. Thus, the examination of p300 expression by IHC could be used as an additional tool in identifying those patients at risk of NPC progression; p300 expression analysis may also be useful in optimizing individual NPC therapy management: favoring a more aggressive regimen in tumors with a high expression of p300.

Several characteristics of p300 suggested that the protein might serve as a tumor suppressor; however, some studies indicated an important role of p300 protein in oncogenic processes [6,23]. In prostate cancer, p300 expression was shown to be linked to proliferation and was found predictive for progression of this cancer [14]. In colon carcinoma, overexpression of p300 was indicative of poor prognosis [19]. Moreover, p300 mRNA levels were observed to correlate with lymph node status in breast cancer [22]. However, p300 protein levels did not show significant correlations with tumor grade or nodal positivity in other study [24,25]. In the present study, we observed that high expression of p300 was associated with an aggressive feature of NPC and was a strong and independent predictor of shorter cancer-specific survival. Considering that the mechanism by which coactivator p300 promotes gene transcription may vary among gene targets, it is not very hard for us to understand that the function of p300 and its underling mechanism(s) to impact cancer progression may lead to this discrepancy, our findings suggest a potential important role of *p300* in the control of NPC cell proliferation, an activity that might be responsible, at least in part, for NPC tumorigenesis and/or progression.

## Conclusions

Our findings provide a basis for the concept that high expression of p300 may play an important role in the acquisition of an aggressive phenotype in NPC, suggesting that the expression of p300, as examined by IHC, will be a promising independent biomarker for shortened survival time of NPC patients.

## Abbreviations

NPC: Nasopharyngeal carcinoma; IHC: Immunohistochemistry; ROC: Receiver operating characteristic; TMA: Tissue microarray.

## Competing interests

The authors declare that they have no competing interests.

## Authors’ contributions

ZWL and TCZ are responsible for the study design. ZWL and XJT performed the experiments and draft the manuscript. LLD and BJT collected the data. XLS, YL, XYS, WJH, and XDL participated in the data analysis and interpretation. All authors read and approved the final manuscript.

## Supplementary Material

Additional file 1 **Figure S1.** Negative control of p300 IHC. Click here for file
