# Supplementary figures and images for: High expression of p300 is linked to aggressive features and poor prognosis of Nasopharyngeal Carcinoma
Source: J Transl Med. 2012 May 30;10:110. doi: 10.1186/1479-5876-10-110 (PMC3484019; doi:10.1186/1479-5876-10-110)

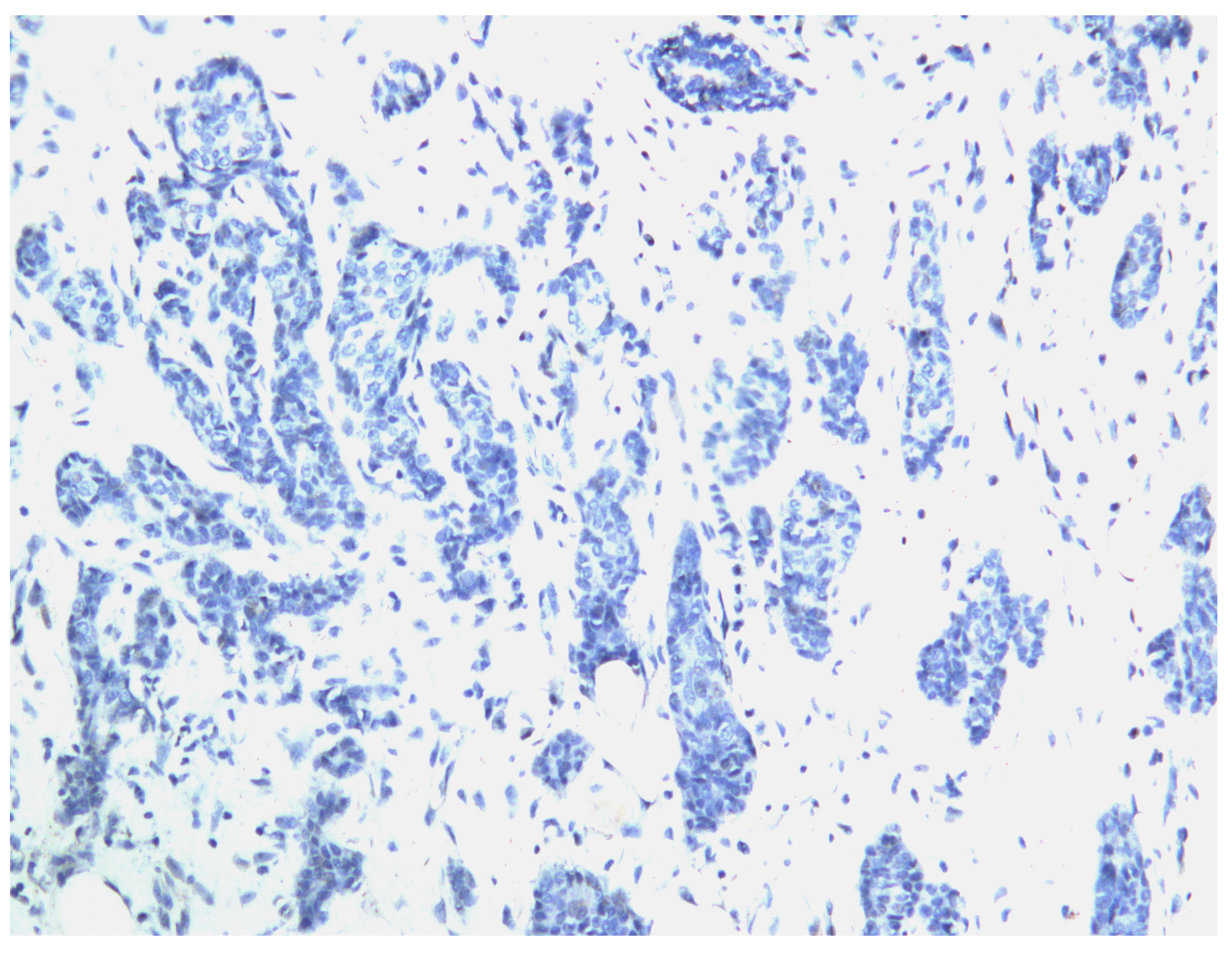

Supplement: Additional file 1 — Figure S1. Negative control of p300 IHC. [file 1479-5876-10-110-S1.tiff]
